# Supplementary material for: Association of short poor work ability measure with increased mortality risk: a prospective multicohort study
Source: BMJ Open. 2022 Dec 22;12(12):e065672. doi: 10.1136/bmjopen-2022-065672 (PMC9791446; doi:10.1136/bmjopen-2022-065672)
Supplement: Supplementary data [file bmjopen-2022-065672supp001.pdf]

Supplementary table 1. Characteristcs of the Mini-Finland - sample

	Total (N=5897)
**Sex**	
Male	2835 (48.1%)
Female	3062 (51.9%)
**Age_years**	
Mean (SD)	45.8 (9.93)
Median [Min, Max]	45.0 [30.0, 64.0]
**Education**	
Lower	3748 (63.6%)
Intermediate	1684 (28.6%)
Higher	459 (7.79%)
**Alcohol_consumption**	
Low	5397 (95.3%)
High	268 (4.73%)
**Current_smoker**	
No	3047 (51.7%)
Yes	2850 (48.3%)
**Body_Mass_Index**	
Mean (SD)	25.7 (4.02)
Median [Min, Max]	25.2 [16.0, 47.8]
**Mental_health_problems**	
No	4420 (93.8%)
Yes	291 (6.18%)
**Poor_self_rated_health**	
No	2965 (52.3%)
Yes	2700 (47.7%)
**Follow_up_time_from_baseline_years**	
Mean (SD)	3.95 (0.373)
Median [Min, Max]	4.00 [0.00824, 4.00]

Supplementary table 2. Characteristcs of the Health2000 - sample

	Total (N=6723)
**Sex**	
Male	3273 (48.7%)
Female	3450 (51.3%)
**ikä 1.7.2000**	
Mean (SD)	41.1 (12.8)
Median [Min, Max]	42.0 [18.0, 64.0]
**Education**	
Lower	1587 (30.5%)
Intermediate	1882 (36.2%)
Higher	1729 (33.3%)
**Alcohol_consumption**	
Low	5079 (92.4%)
High	416 (7.57%)
**Current_smoker**	
No	4419 (66.0%)
Yes	2276 (34.0%)
**BMI: Painoindeksi**	
Mean (SD)	26.0 (4.70)
Median [Min, Max]	25.3 [12.1, 53.8]
**Mental_health_problems**	
No	5233 (83.7%)
Yes	1021 (16.3%)
**Poor_self_rated_health**	
No	4946 (73.9%)
Yes	1749 (26.1%)
**Short_follow_up_time_from_baseline_years**	
Mean (SD)	3.98 (0.229)
Median [Min, Max]	4.00 [0.0165, 4.00]

Supplementary table 3. Characteristcs of the FinHealth - sample

	Total (N=4556)
**Sex**	
Male	2169 (47.6%)
Female	2387 (52.4%)
**Age_years**	
Mean (SD)	46.1 (12.1)
Median [Min, Max]	47.0 [18.4, 65.0]
**Education**	
Lower	465 (10.2%)
Intermediate	1705 (37.5%)
Higher	2371 (52.2%)
**Alcohol_consumption**	
Low	3335 (73.5%)
High	1201 (26.5%)
**Current_smoker**	
No	1725 (72.6%)
Yes	652 (27.4%)
**Body_Mass_Index**	
Mean (SD)	26.9 (4.99)
Median [Min, Max]	26.0 [15.1, 62.1]
**Mental_health_problems**	
No	4108 (92.2%)
Yes	348 (7.81%)
**Poor_self_rated_health**	
No	3282 (72.2%)
Yes	1261 (27.8%)
**Follow_up_time_from_baseline_years**	
Mean (SD)	3.77 (0.196)
Median [Min, Max]	3.79 [0.146, 3.96]

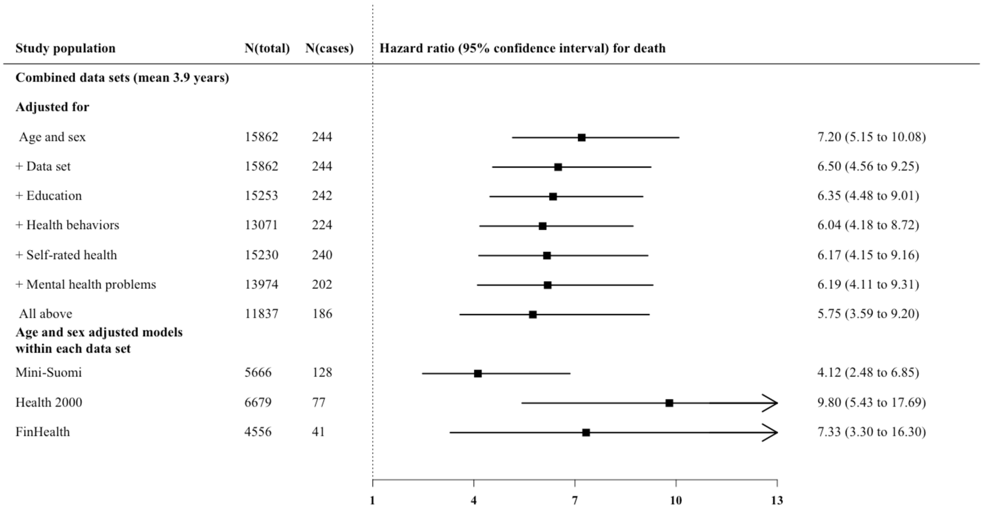

Supplementary figure 1. Associations between poor vs. good workability and mortality risk in pooled and al individual data sets,
