# Supplementary material for: Association of short poor work ability measure with increased mortality risk: a prospective multicohort study
Source: BMJ Open. 2022 Dec 22;12(12):e065672. doi: 10.1136/bmjopen-2022-065672 (PMC9791446; doi:10.1136/bmjopen-2022-065672)
Supplement: Author's manuscript [file bmjopen-2022-065672.draft_revisions.pdf]

BMJ Open

BMJ Open is committed to open peer review. As part of this commitment we make the peer review history of every article we publish publicly available.

When an article is published we post the peer reviewers' comments and the authors' responses online. We also post the versions of the paper that were used during peer review. These are the versions that the peer review comments apply to.

The versions of the paper that follow are the versions that were submitted during the peer review process. They are not the versions of record or the final published versions. They should not be cited or distributed as the published version of this manuscript.

BMJ Open is an open access journal and the full, final, typeset and author-corrected version of record of the manuscript is available on our site with no access controls, subscription charges or pay-per-view fees (http://bmjopen.bmj.com).

If you have any questions on BMJ Open's open peer review process please email info.bmjopen@bmj.com

BMJ Open

Poor work ability as a risk factor for mortality: evidence from three large population-based cohorts

Journal:	*BMJ Open*
Manuscript ID	bmjopen-2022-065672
Article Type:	Original research
Date Submitted by the Author:	15-Jun-2022

Complete List of Authors:	Elovainio, Marko; University of Helsinki; University of Helsinki, Department of Psychology and Logopedics<br>Laaksonen, Mikko; Finnish Centre for Pensions<br>Sakari, Kainulainen; Diaconia University of Applied Sciences<br>Aalto, Anna-Mari; Finnish Institute for Health and Welfare<br>Jääskeläinen, Tuija; Finnish Institute for Health and Welfare, Department of Public Health<br>Rissanen, Harri; Finnish Institute for Health and Welfare<br>Koskinen, Seppo; Finnish Institute for Health and Welfare, Functional capacity and Welfare

Keywords:	EPIDEMIOLOGY, PUBLIC HEALTH, OCCUPATIONAL & INDUSTRIAL MEDICINE

SCHOLARONE™
Manuscripts

I, the Submitting Author has the right to grant and does grant on behalf of all authors of the Work (as defined in the below author licence), an exclusive licence and/or a non-exclusive licence for contributions from authors who are: i) UK Crown employees; ii) where BMJ has agreed a CC-BY licence shall apply, and/or iii) in accordance with the terms applicable for US Federal Government officers or employees acting as part of their official duties; on a worldwide, perpetual, irrevocable, royalty-free basis to BMJ Publishing Group Ltd ("BMJ") its licensees and where the relevant Journal is co-owned by BMJ to the co-owners of the Journal, to publish the Work in this journal and any other BMJ products and to exploit all rights, as set out in our licence.

The Submitting Author accepts and understands that any supply made under these terms is made by BMJ to the Submitting Author unless you are acting as an employee on behalf of your employer or a postgraduate student of an affiliated institution which is paying any applicable article publishing charge ("APC") for Open Access articles. Where the Submitting Author wishes to make the Work available on an Open Access basis (and intends to pay the relevant APC), the terms of reuse of such Open Access shall be governed by a Creative Commons licence – details of these licences and which Creative Commons licence will apply to this Work are set out in our licence referred to above.

Other than as permitted in any relevant BMJ Author's Self Archiving Policies, I confirm this Work has not been accepted for publication elsewhere, is not being considered for publication elsewhere and does not duplicate material already published. I confirm all authors consent to publication of this Work and authorise the granting of this licence.

For peer review only

June 15, 2022

Poor work ability as a risk factor for mortality: evidence from three large population-based cohorts

Marko Elovainio, PhD [1,2,3]

Laaksonen, Mikko[4]

Kainulainen Sakari[5]

Aalto, Anna-Mari[3]

Jääskeläinen, Tuija[3]

Rissanen, Harri[3]

Seppo Koskinen, MD [3]

Short title: Poor work ability and mortality

[1]Research Program Unit, Faculty of Medicine, University of Helsinki, Finland

[2]Department of Psychology and Logopedics, Faculty of Medicine, University of Helsinki, Finland;

[3]Department of Public Health and Welfare, Finnish Institute for Health and Welfare, Helsinki, Finland

[4]Finnish Centre for Pensions, Helsinki, Finland

[5]Diaconia University of Applied Sciences, Helsinki, Finland

Correspondence: Marko Elovainio, University of Helsinki, P.O. Box 9, 00014, Helsinki, Finland, Phone: +358 50 3020621, email: marko.elovainio@helsinki.fi

Word count for main text: 2909 ; Number of tables and figures: 3+2

Keywords: Work, Functional ability, mortality, prediction, population, risk

For peer review only

Abstract

Objectives: To examine the association between a single-item measure of self-rated work ability and all-cause mortality in three large population-based samples collected in 1978-1980, 2000 and 2017.

Setting: A representative sample of the population of Finland.

Participants: The study population comprised 17 178 participants aged 18 to 65 from the population-based Mini-Finland, Health 2000 and FinHealth 2017 cohort studies, pooled together. In all cohorts, self-rated work ability was assessed at baseline (1978-80, 2000-2001 and 2017) using three response alternatives: completely fit, partially disabled (limited work ability) and completely disabled (poor work ability) for work.

Primary and secondary outcome measures: All-cause mortality from national registers. Cox proportional hazards models were adjusted for socioeconomic characteristics, lifestyle factors, self-rated health and mental health problems.

Results: Of the participants, 2219 (13%) were classified as having limited and 991 (5.8%) poor work ability and 246 individuals died during the 4 -year follow-up. The age- and sex-adjusted hazard ratio for mortality risk was 7.20 (95% CI 5.15–10.08) for participants with poor versus good work ability. The excess risk associated with poor work ability was seen in both genders, all age

For peer review only

groups, across different educational levels, self-rated health levels and in those with and without mental health problems. The association was robust to further adjustment for education, health behaviors, self-rated health and mental health problems. In the multivariable analyses, the hazard ratio for mortality among those with poor vs. good work ability was 5.75 (95% CI 3.59–9.20).

Conclusions: Poor self-rated work ability is a strong predictor of increased risk of all-cause mortality and may be a useful survey-measure in predicting severe health outcomes in community-based surveys.

Simple, easy to administer and unambiguous measures of health and functional ability that can predict severe health outcomes and health service use are essential for health surveys and population health monitoring. The single-item question on self-rated health (SRH) has been commonly used as such a measure in psychological research, clinical settings, and general population surveys. SRH is most often measured with a wording: "In general, would you say your health is" with the response items "very good", "quite good", "average", "quite poor" and "very poor". It has repeatedly been shown that SRH associates with physicians' assessments of health (1, 2), chronic disease incidence, physical and cognitive functional limitations, health services use, clinical biomarkers (3-6) and even mortality (7-9).

For peer review only

However, SRH's abstract and latent nature requires a lot of demanding cognitive processes from the study participants, and it has been shown that the meaning of SRH is dependent on psychosocial, socioeconomic and other contextual factors. (10-14). For example, Layes et al. (15) showed that elderly people and those with less income and education seemed to be overly optimistic about their health. Their analysis also indicated a tendency for persons with healthier lifestyles to be overly pessimistic about their health. Similar results have been obtained also elsewhere (16, 17). These results indicate that the assessment of one's general health status is dependent on the reference group and that reference group is not always the same or similarly understood by the researchers and the study participants. As individuals may incorporate numerous factors in their self-perceptions of health, both researchers and studied individuals should have comprehensive and similar conceptualizations of health. In sum, despite the popularity of self-rated health, it may be unclear what the construct captures.

In the rapidly ageing working population work ability is an important dimension of overall health and maintaining work ability is important for countries seeking to keep their citizens in work much longer than previous generations. Poor work ability represents a considerable challenge to the sufficiency of the workforce and to the sustainability of social protection schemes. Promoting the work ability of the working-age population helps people to maintain their health and functional

capacity enabling longer working careers and may also improve the quality of life of the growing population of retirees. The rapid changes in working life over the past few decades, with a shift from physical demands towards cognitive and psychosocial demands, together with changes in the functional capacity, expertise, and attitudes of workers, is reflected in the content of work ability. High quality data on work ability is needed to monitor and promote it. Multiple measures have been developed to measure work ability, but some of the measures such as the work ability index are complicated and inconvenient for use in large-scale surveys (18).

In this study, we tested a self-administered simple measure of work ability as a predictor of overall mortality. The subjective experience of work ability has the advantage over general health evaluation that it has a clear point of reference: subjective work ability is a measure of perceived balance of the demands of the work and the resources of the individual (19). Although the conceptual framework is, of course, much more complicated, the evaluation of ones' capacity to cope with the demands of one's work is probably much less abstract and cognitively challenging than evaluating the overall health status. In this study, we tested whether subjective evaluation of work ability predicts the risk of overall mortality in three large and nationally representative cohorts collected in the 1970s, 2000s and 2010s.

For peer review only

METHODS

The study population

The participants were from three population-based, nationally representative health examination studies: Mini-Finland Health Survey, Health 2000, and FinHealth 2017 (20). In the Mini-Finland Health Survey, 8000 persons (3637 men and 4363 women) aged 30 or over were invited to participate between 1978 and 1980 (21). The sample was representative of the Finnish population (response rate 90%) and the sampling design was stratified two-stage cluster sampling. The survey was carried out in 40 study areas around the country. The final sample in this study was 5897, after excluding persons aged 65 or over. The Health 2000 survey was carried out in 2000–2001. In total, 8028 Finns over the age of 30 and 1894 aged 18 to 29 years were invited to participate. Information about the participants was collected through interviews, an extensive health examination and several questionnaires. A shortened version of the study protocol was used in the age group 18–29 years (22). Two-stage cluster sampling included 15 largest towns and 65 health districts in Finland. The response rate was 85% and the final sample used in this study included 6723 persons aged less than 65 years. The FinHealth 2017 health examination study was carried out in 50 localities in 2017, with the objective of evaluating 10 000 randomly selected persons aged over 18 years in Finland. The study consisted of a physical examination and questionnaires (23). In total, 7 055

For peer review only

people participated, yielding a response rate of 69% and the final sample used in this study covered 4556 persons aged 18–64 years. For the main analyses we combined these three data sets, and restricted the follow-up time to maximum 4 years. A total of 17178 participants alive at the first day of the first phase of each survey were included. Characteristics of the participants in each individual data set are presented in the Supplement tables (**Supplementary table1 - Supplementary table 3**). All three surveys were conducted according to the Declaration of Helsinki. The design, population, and protocol of the individual cohorts have been described in detail elsewhere. (20, 21, 23, 24).

Work ability

In all cohorts, self-rated work ability was assessed at baseline (1978-80, 2000-2001 and 2017) using three response alternatives: completely fit, partially disabled (tai: partially unable) and completely disabled (tai: completely unable) for

In all cohorts, self-rated work ability was assessed at baseline (1978-80, 2000-2001 and 2017) using a single question: “Regardless of whether you are employed or not, please estimate your current work capacity. Are you?” The response alternatives were “completely fit for work”, “partially unable to work” and “completely unable to work”. These categories will be referred as good, limited and poor, respectively.

For peer review only

Mortality

Follow-up for all deaths irrespective of the cause started at inclusion in the study cohort. Information on deaths were obtained from the Causes of Death registry maintained by Statistics Finland and / or from the Death registry from the Digital and population data services agency until the end of 2020.

Assessment of confounders

Potential confounders included common risk factors for poor work ability and mortality. In all sub-cohorts, we assessed sex, age, educational attainment (low, intermediate, high), cigarette smoking (current smoker [yes/no]), alcohol intake frequency (in FinHealth 2017 based on Alcohol Use Disorders Identification Test [low or intermediate vs. high]. In Mini-Finland and Health 2000 based on grams of absolute alcohol from detailed consumption reporting and divided to low and high [in men >280 g/week and in women >210g / week])(25), body mass index (BMI [kg/m2], obesity BMI>=30), self-rated health (good or rather good vs. moderate, rather poor or poor) and mental health problems (In Mini-Finland self-reported mental health problems [No vs. Yes], in Health 2000 based on General Health Questionnaire-12 (26) [classified low and high with a cut-off 4 points] and in FinHealth 2017 based on the 5-item mental health dimension of SF-36 (MHI-5)(27) [classified low and high with a cut-off 52 points]).

For peer review only

Statistical methods

We used Cox proportional hazards models taking into account the survey design to estimate hazard ratios (HRs) for the association of work ability with all-cause mortality. Participants that were 65 years old or older (official retirement age) at baseline were excluded. The follow-up lasted from the study entry of each individual to death or end of the follow-up, whichever came first. The proportional hazards assumption was examined using scaled Schoenfeld residuals.

In the primary analyses, we examined whether having limited or poor work ability predicted death with the following steps. First, we assessed the unadjusted associations separately for men and women, for separate age groups, for separate educational levels and among those with and without poor self-rated health, and with or without mental health problems. Second, we assessed the associations of poor work ability with mortality risk adjusting for (a) age and sex, (b) age, sex, and education (c) age, sex, alcohol consumption, smoking status, BMI and physical activity, (d) age, sex, and self-rated health (e) age, sex and mental health problems and (f) all confounders. The percentage of excess risk explained (PERM) by the confounders was calculated to assess the extent to which the associations of poor work ability with mortality were attributable to differences

For peer review only

between individuals with poor work ability and the other individuals in the level of confounders included in the models.

PERM = ([Hazard ratio $_{\text{(age and sex adjusted)}}$ – hazard ratio $_{\text{(age and sex + X adjusted)}}$]/ [Hazard ratio $_{\text{(age and sex adjusted)}}$ - 1]) x 100.

We also reported the time-dependent ROC /AUC curves for censored survival data for the age and sex adjusted model and then for the model that included age, sex and work ability by using the nearest neighbour estimator for the bivariate distribution function of (X, T), where T represents survival time (28). Finally, we calculated the net reclassification index (NRI) for each variable added to the age and sex adjusted models to evaluate the predictive value of work ability compared to all the other included variables. We conducted all data analyses in R (version 4.1.1).

Role of the funding source

The funders of the study had no role in study design, data collection, data analysis, data interpretation, or writing of the report.

Patient and public involvement

No patient involved.

For peer review only

RESULTS

The primary analysis in the combined cohort contained 17 176 participants (8899 women, 51.8%, and 8277 men, 48.2%). The mean age was 44 years (SD = 11.9 years). Of the participants, 37.1 % had lower, 33.7% intermediate and 29.2% higher educational attainment. Intermediate or poor self-rated health was reported by 35.3% of the participants and 9.8% reported mental health problems (Table 1). We identified 275 deaths (1.6% of the participants). The mean follow-up time from the study entry was 3.9 years (SD = 0.3 years). Of the participants 2219 (13%) were classified as having limited and 991 (5.8%) poor work ability, and 246 individuals died during the follow-up.

Table 1. Characteristics of the participants in the combined data

	**Total (N=17176)**
**Sex**	
Male	8277 (48.2%)
Female	8899 (51.8%)
**Age (years)**	
Mean (SD)	44.0 (11.9)
**Education**	
Lower	5800 (37.1%)
Intermediate	5271 (33.7%)

For peer review only - http://bmjopen.bmj.com/site/about/guidelines.xhtml

Table 1. Characteristics of the participants in the combined data

	Total (N=17176)
Higher	4559 (29.2%)
**Alcohol consumption**	
Low	13811 (88.0%)
High	1885 (12.0%)
**Current smoker**	
No	9191 (61.4%)
Yes	5778 (38.6%)
**Body Mass Index**	
Mean (SD)	26.1 (4.59)
**Mental health problems**	
No	13761 (89.2%)
Yes	1660 (10.8%)
**Moderate or poor self-rated health**	
No	10928 (64.7%)
Yes	5975 (35.3%)
**Follow-up time from baseline (years)**	
Mean (SD)	3.91 (0.293)

The unadjusted hazard ratio for the risk of death among those with limited vs good work ability was 4.58 (95% CI 3.35 - 6.26) and 12.41 (95% CI 9.21–16.72) among those with poor vs. good work ability. This association (Table 2) was evident across sex and age groups, education levels, and

participants with and without poor self-rated health, and with and without mental health problems. However, the association was weaker in men than in women (p-value for interaction for poor work ability was 0.04) (Table 2). No significant interaction effects between cohorts and work ability were found (p-values for interaction with Mini-Finland compared to FinHealth 2017 was 0.21 and with Health 2000 compared to FinHealth 2017 was 0.94). The age and sex adjusted hazard ratio for the risk of death among those with poor vs good work ability was ranged from 4.12 to 9.8 in individual cohorts (**Supplementary figure 1**).

Table 2. Associations between poor work ability and mortality in sub-groups. Numbers are Hazard ratios (HR), 95% confidence intervals (95%CI) and p-values

Study population	*Work ability*	*N*	*Number of deaths*	*HR*	*95% CI*	*p-value*
**All**						
	Limited	16901	246	4.58	3.35 - 6.26	<0.001
	Poor			12.41	9.21 - 16.72	<0.001
**Sex**						
Men	Limited	8140	186	4.92	3.45 - 7.03	<0.001
	Poor			9.74	6.9 - 13.76	<0.001
Women	Limited	8761	60	4.36	2.26 - 8.4	<0.001
	Poor			19.77	10.89 - 35.9	<0.001
**Age-group**						
- 34 years	Limited	3270	13	6.5	1.76 - 24.06	0.01
	Poor			12.29	1.61 - 93.97	0.02
35 - 44 years	Limited	4292	28	4.4	1.72 - 11.22	<0.001
	Poor			9.25	3.39 - 25.27	<0.001
45 - 54 years	Limited	4430	85	3.22	1.87 - 5.55	<0.001
	Poor			11.1	6.78 - 18.16	<0.001
55 - 64 years	Limited	3870	118	2.34	1.46 - 3.73	<0.001

For peer review only

	Poor			5.29	3.38- 8.28	<0.001
**Educational group**						
Lower	Limited	5628	148	3.77	2.49 - 5.7	<0.001
	Poor			8.19	5.5 - 12.21	<0.001
Intermediate	Limited	5210	68	3.7	2.00 - 6.84	<0.001
	Poor			13.33	7.57 - 23.45	<0.001
Higher	Limited	4542	26	3.05	1.03 - 9.01	0.04
	Poor			10.05	3.34 - 30.29	<0.001
**Self-rated heatlh**						
Good	Limited	11179	84	5.23	2.88 - 9.47	<0.001
	Poor			15.72	7.95 - 31.08	<0.001
Moderate or poor	Limited	5699	160	2.99	1.95 - 4.59	<0.001
	Poor			7.93	5.27 - 11.92	<0.001
**Mental health problems**						
Yes	Limited	13730	172	4.89	3.4 - 7.05	<0.001
	Poor			13.41	9.29 - 19.34	<0.001
No	Limited	1655	34	3.1	1.28 - 7.5	0.01
	Poor			6.8	2.93 - 15.76	<0.001

For peer review only

Within the combined data, the age and sex adjusted hazard ratio for the risk of death among those with limited vs. those with good work ability was 3.22 (95% CI 2.30 -4.53) and 7.20 (95% CI 5.15–10.08) among those with poor vs. those with good work ability (Table 3). The association was robust to further adjustment for education, health behaviors, self-rated health and mental health problems. In the multivariable analyses (all above mentioned factors adjusted), the hazard ratio for the risk of death among participants with limited vs. good work-related functional ability was 3.27 (95% CI 2.12–5.05) and 5.75 (95% CI 3.59 – 9.20) among participants with poor vs. good work-related functional ability. The latter risk was decreased by 23% compared to the age and sex

adjusted association. Health behaviors (PERM 19%) and self-rated health (PERM 17%) were the strongest contributors to the association.

Table 3. Associations between poor work ability and mortality (mean follow-up 3.9 years in the combined data set). Numbers are Hazard ratios (HR), 95% confidence intervals (95%CI) and p-values

Adjusted for (in addtion to age and sex)

Models	*Work ability group*	*N*	*No of deaths*	*HR*	*95% CI*	*p-value*
None	Limited	15862	244	3.22	2.30 - 4.53	<0.001
	Poor			7.20	5.15 - 10.08	<0.001
Cohort	Limited	15862	244	3.13	2.22 - 4.41	<0.001
	Poor			6.50	4.56 - 9.25	<0.001
Education	Limited	15253	242	2.96	2.09 - 4.18	<0.001
	Poor			6.35	4.48 - 9.01	<0.001
Health behaviors	Limited	13071	224	2.97	2.07 - 4.26	<0.001
	Poor			6.04	4.18 - 8.72	<0.001
Self-rated health	Limited	15230	240	2.89	1.97 - 4.23	<0.001
	Poor			6.17	4.15 - 9.16	<0.001
Mental health problems	Limited	13974	202	3.05	2.11 - 4.42	<0.001
	Poor			6.19	4.11 - 9.31	<0.001
All above	Limited	11837	186	3.27	2.12 - 5.05	<0.001
	Poor			5.75	3.59 - 9.20	<0.001

The ROC curves displaying sensitivity and specificity of our model including (1) age and sex and (2) age, sex and work ability are presented in Figure 1. Adding work ability improved the performance of the model and suggested that work ability is a good candidate marker for the mortality risk at all time points. The net reclassification indexes are reported in Figure 2. Of all the included variables work ability had the highest NRI. Self-rated health and health behaviors also increased the accuracy of the predicted classification of mortality during the follow-up.

For peer review only

DISCUSSION

The present study using three nationally representative samples suggest that individuals with poor work ability are at a higher risk of mortality than are people with good work ability. This association was observed using a simple, one-item measure of work ability with three response categories. The observed five to six -fold excess risk in the pooled data was not explained by socioeconomic and lifestyle risk factors, self-rated health or mental health problems. The excess risk of mortality in people with poor work ability was also observed in all individual cohorts and thus, our observation may be generalisable over time. Compared to self-rated health, the association between work ability and mortality risk can be considered as robust and large. Our findings support the notion that self-rated health and work ability have only partial overlap with each other and represent different dimensions of health and functional ability. Furthermore, work ability measure clearly improved the prediction of our models compared to other predictors including self-rated health.

For peer review only

It has been shown that poor work ability may predict mortality over relatively long period of time (29), but the majority of research has been conducted on the associations between self-reported work ability and later disability pension or other forms of exit from work (30-33). Most of these previous studies used relatively long and multi-dimensional work ability measures, such as work ability index, although there are studies showing that the predictive validity of one-item measures may be as good as longer and more comprehensive measures (18).

There are, of course, obvious benefits in measuring work ability with long and comprehensive measures that take into account various medical aspects of the individual´s health and functional capacity, the balance between human resources and work demands, the work community, management, the whole psychosocial work environment and environments outside work life (34). The accuracy and comprehensibility required from the work ability measure depends on the purpose for which it is used, and thus, for example, for granting social benefits the measure of work ability and its influencing factors need to be as valid and reliable as possible. Wide definitions and multidimensional measures are needed specifically when we want to find targets for the interventions aiming to enhance work ability at the individual and organizational level (35).

For peer review only

However, using comprehensive and extensive measurements in research is not always feasible or even necessary. When studying a wide variety of factors determining population health, health care use and health care expenditure, it is not possible to measure all potential factors using detailed and long instruments. Thus, short and economic measures with good predictive validity are needed. The one-item work ability measure with three response alternative seems to be a promising option for such a measure, capturing the subjective perception of work ability. This measure is simple, easy to understand and can be used as an indicator of work ability based on the conception people have of their own ability to work.

The present study has a number of strengths. To the best of our knowledge, with more than 17 000 participants from three cohorts, the present study is a large and comprehensive examination of the association between work ability and mortality. Information on mortality was obtained from the national health register with comprehensive recording system for mortality. Thus, the follow-up was virtually complete and independent of active participation in the studies. Potential limitations of the study should also be considered. The response rates in individual cohorts ranged from 90 to 68% but the possibility of selection bias in relation to the investigated exposures and outcomes cannot be

For peer review only

totally excluded. Furthermore, the data were limited to participants aged 65 years and younger at baseline.

Despite these limitations, our findings provide strong population-based evidence to suggest that people with poor self-rated work ability are at increased risk of mortality. Self-rated work ability is a simple and concise measure of health and functional ability with a clear frame of reference and thus it has large potential in epidemiological studies and community-based surveys.

Figure captions

Figure 1. ROC curves of sensitivity and specificity of the model (workability predicting mortality risk) within four follow-up years including age and sex (upper part) and age, sex and work ability (lower part).

Figure 2. Net reclassification indexes (NRI) of all included variables predicting mortality risk (the accuracy of the predicted classification of mortality during the four-year follow-up).

Ethics Approval

The two latter surveys were approved by the Ethics Committees of the Finnish Institute for Health and Welfare and the Helsinki (NPHI 8/99) and Uusimaa hospital region (HUS 37/13/00/12016),

For peer review only

and all participants gave written informed consent. In the 1970's there was no legally prescribed ethical evaluation concerning research work or informed consent procedures, but the general guidelines concerning medicine were also applicable for research. The subjects invited to the Mini-Finland survey were informed on the use of the data for medical research and participation was interpreted as informed consent for research use of the data.

Contributorship statement

ME, ML, SKa and SKo contributed to the concept and design of the study. ME performed all data analyses. ME wrote the first draft of the manuscript. All authors contributed to the interpretation of the results, manuscript revision, and approved the final version of the manuscript. ME is responsible for the overall content as guarantor.

Competing interests

The authors declare no competing interests.

Funding

The present study was supported by the Academy of Finland (339390 to ME). The funding bodies had no role in any stage of the research process.

Data sharing statement

For peer review only

Data used in the current study may be obtained from Statistics Finland, and the Finnish Institute for Health and Welfare. Restrictions apply to the availability of these data, which were used under license for this study. For information on accessing the data see: www.stat.fi, and www.thl.fi.

For peer review only

References

1. Maddox GL. Some correlates of differences in self-assessment of health status among the elderly. *Journal of gerontology* 1962;17:180-5.
2. LaRue A, Bank L, Jarvik L, et al. Health in old age: how do physicians' ratings and self-ratings compare? *Journal of gerontology* 1979;34(5):687-91.
3. Pijls LT, Feskens EJ, Kromhout D. Self-rated health, mortality, and chronic diseases in elderly men. The Zutphen Study, 1985-1990. *Am J Epidemiol* 1993;138(10):840-8.
4. Idler EL, Russell LB, Davis D. Survival, functional limitations, and self-rated health in the NHANES I Epidemiologic Follow-up Study, 1992. First National Health and Nutrition Examination Survey. *Am J Epidemiol* 2000;152(9):874-83.
5. Haseli-Mashhadi N, Pan A, Ye X, et al. Self-Rated Health in middle-aged and elderly Chinese: distribution, determinants and associations with cardio-metabolic risk factors. *BMC Public Health* 2009;9:368.
6. Hayes AJ, Clarke PM, Glasziou PG, et al. Can self-rated health scores be used for risk prediction in patients with type 2 diabetes? *Diabetes Care* 2008;31(4):795-7.
7. Idler EL, Benyamini Y. Self-rated health and mortality: a review of twenty-seven community studies. *J Health Soc Behav* 1997;38(1):21-37.
8. Idler EL, Kasl S. Health perceptions and survival: do global evaluations of health status really predict mortality? *Journal of gerontology* 1991;46(2):S55-65.
9. Idler EL, Kasl SV, Lemke JH. Self-evaluated health and mortality among the elderly in New Haven, Connecticut, and Iowa and Washington counties, Iowa, 1982-1986. *Am J Epidemiol* 1990;131(1):91-103.
10. Delpierre C, Lauwers-Cances V, Datta GD, et al. Impact of social position on the effect of cardiovascular risk factors on self-rated health. *Am J Public Health* 2009;99(7):1278-84.
11. Singh-Manoux A, Martikainen P, Ferrie J, et al. What does self rated health measure? Results from the British Whitehall II and French Gazel cohort studies. *J Epidemiol Community Health* 2006;60(4):364-72.
12. Benyamini Y, Idler EL, Leventhal H, et al. Positive affect and function as influences on self-assessments of health: expanding our view beyond illness and disability. *The journals of gerontology Series B, Psychological sciences and social sciences* 2000;55(2):P107-16.
13. Benyamini Y, Leventhal EA, Leventhal H. Gender differences in processing information for making self-assessments of health. *Psychosom Med* 2000;62(3):354-64.
14. Manderbacka K. Examining what self-rated health question is understood to mean by respondents. *Scand J Soc Med* 1998;26(2):145-53.
15. Layes A, Asada Y, Kepart G. Whiners and deniers - what does self-rated health measure? *Soc Sci Med* 2012;75(1):1-9.
16. Dowd JB, Zajacova A. Does the predictive power of self-rated health for subsequent mortality risk vary by socioeconomic status in the US? *Int J Epidemiol* 2007;36(6):1214-21.
17. Dowd JB, Zajacova A. Does self-rated health mean the same thing across socioeconomic groups? Evidence from biomarker data. *Ann Epidemiol* 2010;20(10):743-9.

For peer review only

18. El Fassi M, Bocquet V, Majery N, et al. Work ability assessment in a worker population: comparison and determinants of Work Ability Index and Work Ability score. *BMC Public Health* 2013;13:305.
19. Lederer V, Loisel P, Rivard M, et al. Exploring the diversity of conceptualizations of work (dis)ability: a scoping review of published definitions. *J Occup Rehabil* 2014;24(2):242-67.
20. Aromaa A, Heliövaara M, Knekt P, et al. *National Health Examination Surveys in Research*. Helsinki: THL; 2019. : https://urn.fi/URN:ISBN:978-952-343-328-1
21. Heliovaara M, Aromaa A, Klaukka T, et al. Reliability and validity of interview data on chronic diseases. The Mini-Finland Health Survey. *J Clin Epidemiol* 1993;46(2):181-91.
22. Heistaro S. *Methodology Report. Health 2000 survey. In Publications of the National Public Health Institute, KTL (ed. N. P. H. Institute).* Helsinki: the National Public Health Institute; 2008 .
23. Borodulin K, Sääksjärvi K eds. FinHealth 2017 Study – Methods. Helsinki: THL, 2019. https://urn.fi/URN:ISBN:978-952-343-449-3
24. Aromaa A, Koskinen S. *Health and functional capacity in Finland. Baseline results of the Health 2000 health examination survey..* Helsinki: National Public Health Institute B3; 2002.
25. Virtanen M, Jokela M, Nyberg ST, et al. Long working hours and alcohol use: systematic review and meta-analysis of published studies and unpublished individual participant data. *BMJ* 2015;350:g7772.
26. Goldberg DP, Gater R, Sartorius N, et al. The validity of two versions of the GHQ in the WHO study of mental illness in general health care. *Psychological Medicine* 1997;27(1):191-7.
27. McCabe CJ, Thomas KJ, Brazier JE, et al. Measuring the mental health status of a population: a comparison of the GHQ-12 and the SF-36 (MHI-5). *Br J Psychiatry* 1996;169(4):516-21.
28. Akritas MG. Nearest-Neighbor Estimation of a Bivariate Distribution under Random Censoring. *Annals of Statistics* 1994;22(3):1299-327.
29. von Bonsdorff. M., Seitsamo J, Ilmarinen J, et al. Work ability in midlife as a predictor of mortality and disability in later life: a 28-year prospective follow-up study. *CMAJ* 2011;184:E235-42.
30. Kinnunen U, Natti J. Work ability score and future work ability as predictors of register-based disability pension and long-term sickness absence: A three-year follow-up study. *Scand J Public Health* 2018;46(3):321-30.
31. Bethge M, Mattukat K, Fauser D, et al. Rehabilitation access and effectiveness for persons with back pain: the protocol of a cohort study (REHAB-BP, DRKS00011554). *BMC Public Health* 2017;18(1):22.
32. Jaaskelainen A, Kausto J, Seitsamo J, et al. Work ability index and perceived work ability as predictors of disability pension: a prospective study among Finnish municipal employees. *Scand J Work Environ Health* 2016;42(6):490-9.
33. Alavinia SM, de Boer AG, van Duivenbooden JC, et al. Determinants of work ability and its predictive value for disability. *Occup Med (Lond)* 2009;59(1):32-7.
34. Ilmarinen J, Tuomi K, Klockars M. Changes in the work ability of active employees over an 11-year period. *Scandinavian Journal of Work, Environment & Health* 1997;23 Suppl 1:49-57.
35. Tuomi K, Huuhtanen P, Nykyri E, et al. Promotion of work ability, the quality of work and retirement. *Occup Med (Lond)* 2001;51(5):318-24.

For peer review only

For peer review only

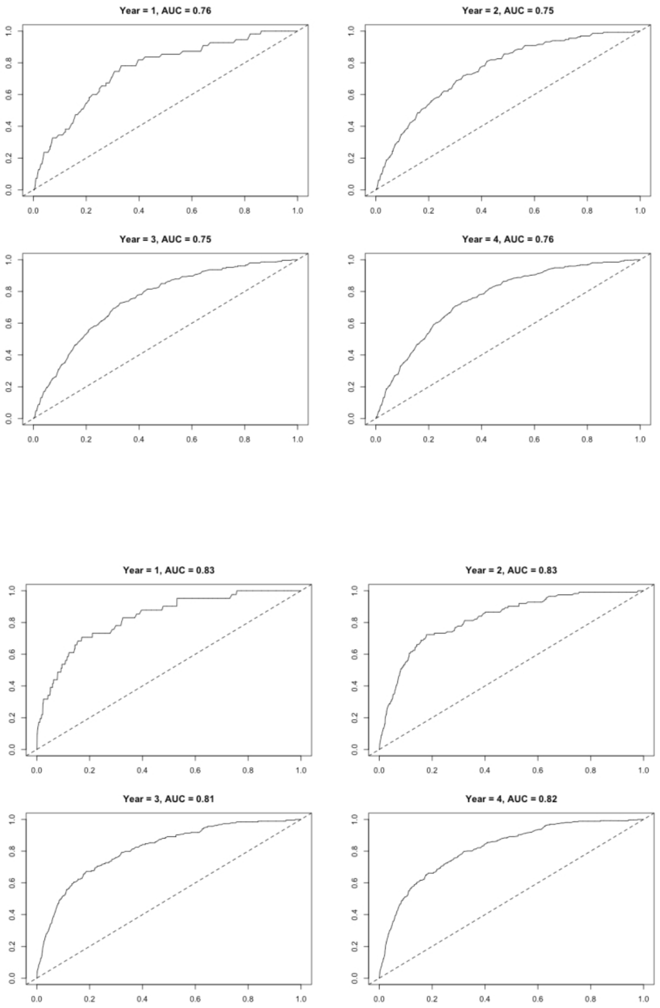

Figure 1. ROC curves of sensitivity and specificity of the model (workability predicting mortality risk) within four follow-up years including age and sex (first row columns) and age, sex and work ability (second row columns).

335x452mm (118 x 118 DPI)

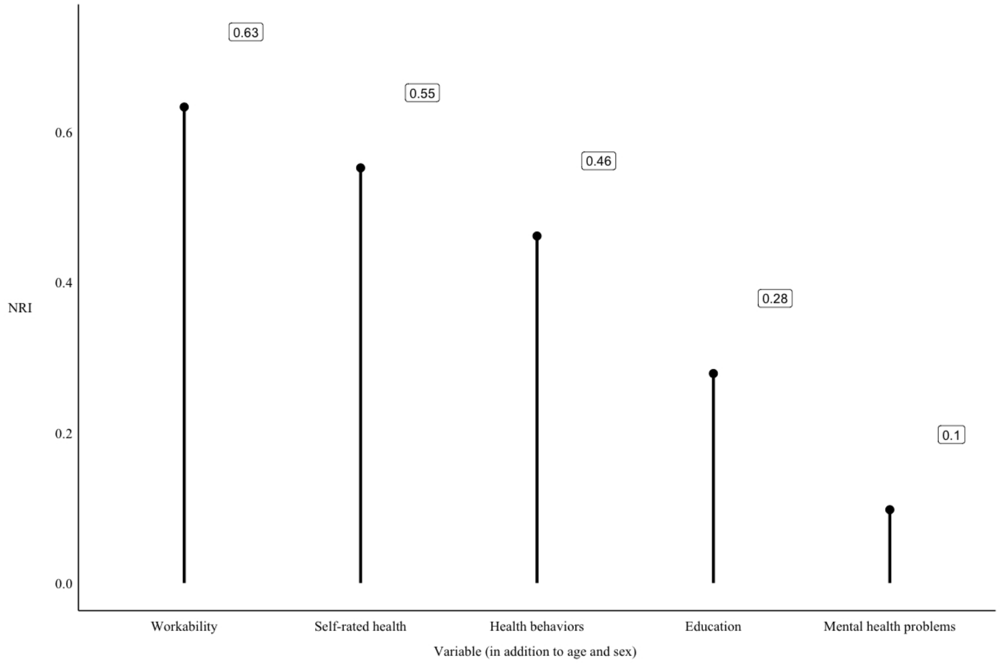

Figure 2. Net reclassification indexes (NRI) of all included variables predicting mortality risk (the accuracy of the predicted classification of mortality during the four-year follow-up).

423x282mm (72 x 72 DPI)

Supplementary table 1. Characteristcs of the Mini-Finland - sample

	Total (N=5897)
**Sex**	
Male	2835 (48.1%)
Female	3062 (51.9%)
**Age_years**	
Mean (SD)	45.8 (9.93)
Median [Min, Max]	45.0 [30.0, 64.0]
**Education**	
Lower	3748 (63.6%)
Intermediate	1684 (28.6%)
Higher	459 (7.79%)
**Alcohol_consumption**	
Low	5397 (95.3%)
High	268 (4.73%)
**Current_smoker**	
No	3047 (51.7%)
Yes	2850 (48.3%)
**Body_Mass_Index**	
Mean (SD)	25.7 (4.02)
Median [Min, Max]	25.2 [16.0, 47.8]
**Mental_health_problems**	
No	4420 (93.8%)
Yes	291 (6.18%)
**Poor_self_rated_health**	
No	2965 (52.3%)
Yes	2700 (47.7%)
**Follow_up_time_from_baseline_years**	
Mean (SD)	3.95 (0.373)
Median [Min, Max]	4.00 [0.00824, 4.00]

Supplementary table 2. Characteristcs of the Health2000 - sample

	Total (N=6723)
**Sex**	
Male	3273 (48.7%)
Female	3450 (51.3%)
**ikä 1.7.2000**	
Mean (SD)	41.1 (12.8)
Median [Min, Max]	42.0 [18.0, 64.0]
**Education**	
Lower	1587 (30.5%)
Intermediate	1882 (36.2%)
Higher	1729 (33.3%)
**Alcohol_consumption**	
Low	5079 (92.4%)
High	416 (7.57%)
**Current_smoker**	
No	4419 (66.0%)
Yes	2276 (34.0%)
**BMI: Painoindeksi**	
Mean (SD)	26.0 (4.70)
Median [Min, Max]	25.3 [12.1, 53.8]
**Mental_health_problems**	
No	5233 (83.7%)
Yes	1021 (16.3%)
**Poor_self_rated_health**	
No	4946 (73.9%)
Yes	1749 (26.1%)
**Short_follow_up_time_from_baseline_years**	
Mean (SD)	3.98 (0.229)
Median [Min, Max]	4.00 [0.0165, 4.00]

Supplementary table 3. Characteristcs of the FinHealth - sample

	Total (N=4556)
**Sex**	
Male	2169 (47.6%)
Female	2387 (52.4%)
**Age_years**	
Mean (SD)	46.1 (12.1)
Median [Min, Max]	47.0 [18.4, 65.0]
**Education**	
Lower	465 (10.2%)
Intermediate	1705 (37.5%)
Higher	2371 (52.2%)
**Alcohol_consumption**	
Low	3335 (73.5%)
High	1201 (26.5%)
**Current_smoker**	
No	1725 (72.6%)
Yes	652 (27.4%)
**Body_Mass_Index**	
Mean (SD)	26.9 (4.99)
Median [Min, Max]	26.0 [15.1, 62.1]
**Mental_health_problems**	
No	4108 (92.2%)
Yes	348 (7.81%)
**Poor_self_rated_health**	
No	3282 (72.2%)
Yes	1261 (27.8%)
**Follow_up_time_from_baseline_years**	
Mean (SD)	3.77 (0.196)
Median [Min, Max]	3.79 [0.146, 3.96]

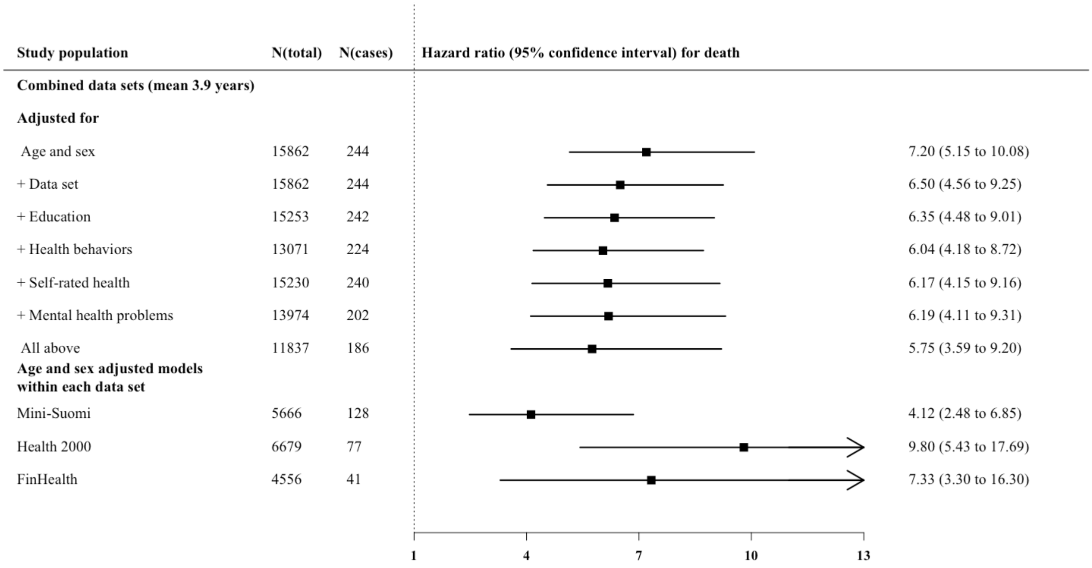

Supplementary figure 1. Associations between poor vs. good workability and mortality risk in pooled and al individual data sets,

For peer review only

BMJ Open

Association of short poor work ability -measure with increased mortality risk: a prospective multicohort study

Journal:	*BMJ Open*
Manuscript ID	bmjopen-2022-065672.R1
Article Type:	Original research
Date Submitted by the Author:	03-Nov-2022
Complete List of Authors:	Elovainio, Marko; University of Helsinki; University of Helsinki, Department of Psychology and Logopedics<br>Laaksonen, Mikko; Finnish Centre for Pensions<br>Sakari, Kainulainen; Diaconia University of Applied Sciences<br>Aalto, Anna-Mari; Finnish Institute for Health and Welfare<br>Jääskeläinen, Tuija; Finnish Institute for Health and Welfare, Department of Public Health<br>Rissanen, Harri; Finnish Institute for Health and Welfare<br>Koskinen, Seppo; Finnish Institute for Health and Welfare, Functional capacity and Welfare
**Primary Subject Heading**:	Occupational and environmental medicine
Secondary Subject Heading:	Occupational and environmental medicine
Keywords:	EPIDEMIOLOGY, PUBLIC HEALTH, OCCUPATIONAL & INDUSTRIAL MEDICINE

SCHOLARONE™
Manuscripts

I, the Submitting Author has the right to grant and does grant on behalf of all authors of the Work (as defined in the below author licence), an exclusive licence and/or a non-exclusive licence for contributions from authors who are: i) UK Crown employees; ii) where BMJ has agreed a CC-BY licence shall apply, and/or iii) in accordance with the terms applicable for US Federal Government officers or employees acting as part of their official duties; on a worldwide, perpetual, irrevocable, royalty-free basis to BMJ Publishing Group Ltd ("BMJ") its licensees and where the relevant Journal is co-owned by BMJ to the co-owners of the Journal, to publish the Work in this journal and any other BMJ products and to exploit all rights, as set out in our licence.

The Submitting Author accepts and understands that any supply made under these terms is made by BMJ to the Submitting Author unless you are acting as an employee on behalf of your employer or a postgraduate student of an affiliated institution which is paying any applicable article publishing charge ("APC") for Open Access articles. Where the Submitting Author wishes to make the Work available on an Open Access basis (and intends to pay the relevant APC), the terms of reuse of such Open Access shall be governed by a Creative Commons licence – details of these licences and which Creative Commons licence will apply to this Work are set out in our licence referred to above.

Other than as permitted in any relevant BMJ Author's Self Archiving Policies, I confirm this Work has not been accepted for publication elsewhere, is not being considered for publication elsewhere and does not duplicate material already published. I confirm all authors consent to publication of this Work and authorise the granting of this licence.

For peer review only

November 2, 2022

Association of short poor work ability -measure with increased mortality risk: a prospective multicohort study

Marko Elovainio, PhD [1,2,3]
Laaksonen, Mikko[4]
Kainulainen Sakari[5]
Aalto, Anna-Mari[3]
Jääskeläinen, Tuija[3]
Rissanen, Harri[3]
Seppo Koskinen, MD [3]

Short title: Poor work ability and mortality

[1]Research Program Unit, Faculty of Medicine, University of Helsinki, Finland
[2]Department of Psychology and Logopedics, Faculty of Medicine, University of Helsinki, Finland;
[3]Department of Public Health and Welfare, Finnish Institute for Health and Welfare, Helsinki, Finland
[4]Finnish Centre for Pensions, Helsinki, Finland
[5]Diaconia University of Applied Sciences, Helsinki, Finland

Correspondence: Marko Elovainio, University of Helsinki, P.O. Box 9, 00014, Helsinki, Finland, Phone: +358 50 3020621, email: marko.elovainio@helsinki.fi

Word count for main text: 2909 ; Number of tables and figures: 3+2

Keywords: Work, Functional ability, mortality, prediction, population, risk

For peer review only

For peer review only

Abstract

Objectives: To examine whether a single-item measure of self-rated work ability predict all-cause mortality in three large population-based samples collected in 1978-1980, 2000 and 2017.

Setting: A representative sample of the population of Finland.

Participants: The study population comprised 17 178 participants aged 18 to 65 from the population-based Mini-Finland, Health 2000 and FinHealth 2017 cohort studies, pooled together. In all cohorts, self-rated work ability was assessed at baseline (1978-80, 2000-2001 and 2017) using three response alternatives: completely fit (good work ability), partially disabled (limited work ability) and completely disabled (poor work ability) for work.

Primary and secondary outcome measures: All-cause mortality from national registers. Cox proportional hazards models were adjusted for socioeconomic characteristics, lifestyle factors, self-rated health and mental health problems.

Results: Of the participants, 2219 (13%) were classified as having limited and 991 (5.8%) poor work ability and 246 individuals died during the 4 -year follow-up. The age- and sex-adjusted hazard ratio for mortality risk was 7.20 (95% CI 5.15–10.08) for participants with poor versus good work ability and 3.22 (95% CI 2.30–4.43) for participants with limited versus good work ability. The excess risk associated with poor work ability was seen in both genders, all age groups, across

For peer review only

different educational levels, self-rated health levels and in those with and without mental health problems. The associations were robust to further adjustment for education, health behaviors, self-rated health and mental health problems. In the multivariable analyses, the hazard ratio for mortality among those with poor vs. good work ability was 5.75 (95% CI 3.59–9.20).

Conclusions: One-item poor self-rated work ability -measure is a strong predictor of increased risk of all-cause mortality and may be a useful survey-measure in predicting severe health outcomes in community-based surveys.

For peer review only

Strengths and limitations of this study

- Our observational study used three high-quality population-based datasets from three different historical contexts pooled together.
- A wide range of important confounders including sociodemographic factors, lifestyle, health status and mental health problems .were considered in the analyses
- The study is limited by being observational rather than interventional

For peer review only

Introduction

In the rapidly ageing working population work ability is an important dimension of overall health and maintaining work ability is important for countries seeking to keep their citizens in work much longer than previous generations (1). The rapid changes in working life over the past few decades, with a shift from physical demands towards cognitive and psychosocial demands, together with changes in the functional capacity, expertise, and attitudes of workers, is reflected in the content of work ability. Poor work ability represents a considerable challenge to the sufficiency of the workforce and to the sustainability of social protection schemes. Promoting the work ability of the working-age population helps people to maintain their health and functional capacity enabling longer working careers and may also improve the quality of life of the growing population of retirees.

There are efficient risk-adjusted, stepped care models and interventions for work disability management (2), but these models and interventions need to be focused on those that really need and benefit from them. The primary precondition for correctly focused interventions is easily administered and reliable measure of work disability. Currently the identification of workers in increased risk of work disability is based on the duration of sickness absence (3) Sickness absence is a good predictor of ill health and permanent work disability (4), but only a small proportion of

For peer review only

those with even long -term sickness absences really need sustained support to maintain work ability (5). Thus, there is a clear need of a simple, easy to administer and unambiguous measures of work-related health and functional ability that can predict severe health outcomes and health service use. Such measures are also essential for health surveys and population health monitoring.

One of the most widely used one is the Work Ability Index, a self-report survey screening instrument (6, 7). The questionnaire assesses with 7 dimensions and 16 questions the degree to which workers consider their state of health adequate to cope with their job demands. The continuous scores can be categorized into groups that reflect different levels of need of support. Although widely used and reliable (1), the work ability index is still rather complicated and inconvenient for use in large-scale surveys (8). A more simple, single-item question on self-rated health (SRH) has been commonly used as a measure of health status in psychological research, clinical settings, and general population surveys. It has also repeatedly been shown that SRH associates with physicians' assessments of health (9, 10), chronic disease incidence, physical and cognitive functional limitations, health services use, clinical biomarkers (11-14) and even mortality (15-17).

In this study, we tested a self-administered one-item measure of work ability as a predictor of overall mortality. The subjective experience of work ability has the advantage over general health

For peer review only

evaluation that it has a clear point of reference: subjective work ability is a measure of perceived balance of the demands of the work and the resources of the individual (18). Although the conceptual framework is, of course, much more complicated, the evaluation of ones' capacity to cope with the demands of one's work is probably much less abstract and cognitively challenging than evaluating the overall health status. We tested whether subjective evaluation of work ability predicts the risk of overall mortality in three large and nationally representative cohorts collected in the 1970s, 2000s and 2010s. We also tested whether the one-item work ability measure predict mortality risk as well as and independently of the one-item self-rated health measure.

METHODS

The study population

The participants were from three population-based, nationally representative health examination studies: Mini-Finland Health Survey, Health 2000, and FinHealth 2017 (19). In the Mini-Finland Health Survey, 8000 persons (3637 men and 4363 women) aged 30 or over were invited to participate between 1978 and 1980 (20). The sample was representative of the Finnish population (response rate 90%) and the sampling design was stratified two-stage cluster sampling. The survey was carried out in 40 study areas around the country. The final sample in this study was 5897, after

For peer review only - http://bmjopen.bmj.com/site/about/guidelines.xhtml

excluding persons aged 65 or over. The Health 2000 survey was carried out in 2000–2001. In total, 8028 Finns over the age of 30 and 1894 aged 18 to 29 years were invited to participate. Information about the participants was collected through interviews, an extensive health examination and several questionnaires. A shortened version of the study protocol was used in the age group 18–29 years (21). Two-stage cluster sampling included 15 largest towns and 65 health districts in Finland. The response rate was 85% and the final sample used in this study included 6723 persons aged less than 65 years. The FinHealth 2017 health examination study was carried out in 50 localities in 2017, with the objective of evaluating 10 000 randomly selected persons aged over 18 years in Finland. The study consisted of a physical examination and questionnaires (22). In total, 7 055 people participated, yielding a response rate of 69% and the final sample used in this study covered 4556 persons aged 18–64 years. For the main analyses we combined these three data sets. We restricted the follow-up time to maximum 4 years, because that was the maximum follow up time in the lates (FinHealth2017) cohort to make data sets comparable. A total of 17178 participants alive at the first day of the first phase of each survey were included. Characteristics of the participants in each individual data set are presented in the Supplement tables (**Supplementary table1 - Supplementary table 3**). All three surveys were conducted according to the Declaration of Helsinki. The design, population, and protocol of the individual cohorts have been described in detail elsewhere. (19, 20, 22, 23).

For peer review only

Work ability

In all cohorts, self-rated work ability was assessed at baseline (1978-80, 2000-2001 and 2017) using a single question: "Regardless of whether you are employed or not, please estimate your current work capacity. Are you?" The response alternatives were "completely fit for work", "partially unable to work" and "completely unable to work". These categories will be referred as good, limited and poor, respectively.

Mortality

Follow-up for all deaths irrespective of the cause started at inclusion in the study cohort. Information on deaths and the dates of death were obtained from the Causes of Death registry maintained by Statistics Finland and the Death registry from the Digital and population data services agency until the end of 2020. The Causes of Death registry includes information of all deaths in Finland.

Assessment of confounders

For peer review only

Potential confounders included common risk factors for poor work ability and mortality. In all sub-cohorts, we assessed sex, age, educational attainment (low, intermediate, high), cigarette smoking (current smoker [yes/no]), alcohol intake frequency (in FinHealth 2017 based on Alcohol Use Disorders Identification Test [low or intermediate vs. high]. In Mini-Finland and Health 2000 based on grams of absolute alcohol from detailed consumption reporting and divided to low and high [in men >280 g/week and in women >210g / week])(24), body mass index (BMI [kg/m2], obesity BMI>=30), self-rated health (good or rather good vs. moderate, rather poor or poor) and mental health problems (In Mini-Finland self-reported mental health problems [No vs. Yes], in Health 2000 based on General Health Questionnaire-12 (25) [classified low and high with a cut-off 4 points] and in FinHealth 2017 based on the 5-item mental health dimension of SF-36 (MHI-5)(26) [classified low and high with a cut-off 52 points]).

Statistical methods

We used Cox proportional hazards models taking into account the survey design to estimate hazard ratios (HRs) for the association of work ability with all-cause mortality. Participants that were 65 years old or older (official retirement age) at baseline were excluded. The follow-up lasted from the

For peer review only - http://bmjopen.bmj.com/site/about/guidelines.xhtml

study entry of each individual to death or end of the follow-up, whichever came first. The proportional hazards assumption was examined using scaled Schoenfeld residuals.

In the primary analyses, we examined whether having limited or poor work ability predicted death with the following steps. First, we assessed the unadjusted associations separately for men and women, for separate age groups, for separate educational levels and among those with and without poor self-rated health, and with or without mental health problems. Second, we assessed the associations of poor work ability with mortality risk adjusting for (a) age and sex, (b) age, sex, and education (c) age, sex, alcohol consumption, smoking status, BMI and physical activity, (d) age, sex, and self-rated health (e) age, sex and mental health problems and (f) all confounders. The percentage of excess risk explained (PERM) by the confounders was calculated to assess the extent to which the associations of poor work ability with mortality were attributable to differences between individuals with poor work ability and the other individuals in the level of confounders included in the models.

PERM = ([Hazard ratio $_{\text{(age and sex adjusted)}}$ – hazard ratio $_{\text{(age and sex + X adjusted)}}$]/ [Hazard ratio $_{\text{(age and sex adjusted)}}$ - 1]) x 100.

For peer review only

We also reported the time-dependent ROC /AUC curves for censored survival data for the age and sex adjusted model and then for the model that included age, sex and work ability by using the nearest neighbour estimator for the bivariate distribution function of (X, T), where T represents survival time (27). Finally, we calculated the net reclassification index (NRI) for each variable added to the age and sex adjusted models to evaluate the predictive value of work ability compared to all the other included variables. We conducted all data analyses in R (version 4.1.1).

Role of the funding source

The funders of the study had no role in study design, data collection, data analysis, data interpretation, or writing of the report.

Patient and public involvement

No patient involved.

For peer review only

RESULTS

The primary analysis in the combined cohort contained 17 176 participants (8899 women, 51.8%, and 8277 men, 48.2%). The mean age was 44 years (SD = 11.9 years). Of the participants, 37.1 % had lower, 33.7% intermediate and 29.2% higher educational attainment. Intermediate or poor self-

rated health was reported by 35.3% of the participants and 9.8% reported mental health problems (Table 1). We identified 275 deaths (1.6% of the participants). The mean follow-up time from the study entry was 3.9 years (SD = 0.3 years). Of the participants 2219 (13%) were classified as having limited and 991 (5.8%) poor work ability, and 246 individuals died during the follow-up.

Table 1. Characteristics of the participants in the combined data

	Total (N=17176)
**Sex**	
Male	8277 (48.2%)
Female	8899 (51.8%)
**Age (years)**	
Mean (SD)	44.0 (11.9)
**Education**	
Lower	5800 (37.1%)
Intermediate	5271 (33.7%)
Higher	4559 (29.2%)
**Alcohol consumption**	
Low	13811 (88.0%)
High	1885 (12.0%)
**Current smoker**	
No	9191 (61.4%)
Yes	5778 (38.6%)
**Body Mass Index**	
Mean (SD)	26.1 (4.59)

Table 1. Characteristics of the participants in the combined data

	Total (N=17176)
**Mental health problems**	
No	13761 (89.2%)
Yes	1660 (10.8%)
**Moderate or poor self-rated health**	
No	10928 (64.7%)
Yes	5975 (35.3%)
**Follow-up time from baseline (years)**	
Mean (SD)	3.91 (0.293)

The unadjusted hazard ratio for the risk of death among those with limited vs good work ability was 4.58 (95% CI 3.35 - 6.26) and 12.41 (95% CI 9.21–16.72) among those with poor vs. good work ability. This association (Table 2) was evident across sex and age groups, education levels, and participants with and without poor self-rated health, and with and without mental health problems. However, the association was weaker in men than in women (p-value for interaction for poor work ability was 0.04) (Table 2). No significant interaction effects between cohorts and work ability were found (p-values for interaction with Mini-Finland compared to FinHealth 2017 was 0.21 and with Health 2000 compared to FinHealth 2017 was 0.94). The age and sex adjusted hazard ratio for the

For peer review only

risk of death among those with poor vs good work ability was ranged from 4.12 to 9.8 in individual cohorts (**Supplementary figure 1**).

Table 2. Associations between poor work ability and mortality in sub-groups. Numbers are Hazard ratios (HR), 95% confidence intervals (95%CI) and p-values

Study population	*Work ability*	*N*	*Number of deaths*	*HR*	*95% CI*	*p-value*
**All**						
	Limited	16901	246	4.58	3.35 - 6.26	<0.001
	Poor			12.41	9.21 - 16.72	<0.001
**Sex**						
Men	Limited	8140	186	4.92	3.45 - 7.03	<0.001
	Poor			9.74	6.9 - 13.76	<0.001
Women	Limited	8761	60	4.36	2.26 - 8.4	<0.001
	Poor			19.77	10.89 - 35.9	<0.001
**Age-group**						
- 34 years	Limited	3270	13	6.5	1.76 - 24.06	0.01
	Poor			12.29	1.61 - 93.97	0.02
35 - 44 years	Limited	4292	28	4.4	1.72 - 11.22	<0.001
	Poor			9.25	3.39 - 25.27	<0.001
45 - 54 years	Limited	4430	85	3.22	1.87 - 5.55	<0.001
	Poor			11.1	6.78 - 18.16	<0.001
55 - 64 years	Limited	3870	118	2.34	1.46 - 3.73	<0.001
	Poor			5.29	3.38- 8.28	<0.001
**Educational group**						
Lower	Limited	5628	148	3.77	2.49 - 5.7	<0.001
	Poor			8.19	5.5 - 12.21	<0.001
Intermediate	Limited	5210	68	3.7	2.00 - 6.84	<0.001
	Poor			13.33	7.57 - 23.45	<0.001
Higher	Limited	4542	26	3.05	1.03 - 9.01	0.04
	Poor			10.05	3.34 - 30.29	<0.001
**Self-rated heatlh**						
Good	Limited	11179	84	5.23	2.88 - 9.47	<0.001

For peer review only

	Poor			15.72	7.95 - 31.08	<0.001
Moderate or poor	Limited	5699	160	2.99	1.95 - 4.59	<0.001
	Poor			7.93	5.27 - 11.92	<0.001
**Mental health problems**						
Yes	Limited	13730	172	4.89	3.4 - 7.05	<0.001
	Poor			13.41	9.29 - 19.34	<0.001
No	Limited	1655	34	3.1	1.28 - 7.5	0.01
	Poor			6.8	2.93 - 15.76	<0.001

For peer review only

Within the combined data, the age and sex adjusted hazard ratio for the risk of death among those with limited vs. those with good work ability was 3.22 (95% CI 2.30 -4.53) and 7.20 (95% CI 5.15–10.08) among those with poor vs. those with good work ability (Table 3). The association was robust to further adjustment for education, health behaviors, self-rated health and mental health problems. In the multivariable analyses (all above mentioned factors adjusted), the hazard ratio for the risk of death among participants with limited vs. good work-related functional ability was 3.27 (95% CI 2.12–5.05) and 5.75 (95% CI 3.59 – 9.20) among participants with poor vs. good work-related functional ability. The latter risk was decreased by 23% compared to the age and sex adjusted association. Health behaviors (PERM 19%) and self-rated health (PERM 17%) were the strongest contributors to the association.

Table 3. Associations between poor work ability and mortality (mean follow-up 3.9 years in the combined data set). Numbers are Hazard ratios (HR), 95% confidence intervals (95%CI) and p-values

Adjusted for (in addtion to age and sex)

Models	*Work ability group*	*N*	*No of deaths*	*HR*	*95% CI*	*p-value*

None	Limited	15862	244	3.22	2.30 - 4.53	<0.001
	Poor			7.20	5.15 - 10.08	<0.001
Cohort	Limited	15862	244	3.13	2.22 - 4.41	<0.001
	Poor			6.50	4.56 - 9.25	<0.001
Education	Limited	15253	242	2.96	2.09 - 4.18	<0.001
	Poor			6.35	4.48 - 9.01	<0.001
Health behaviors	Limited	13071	224	2.97	2.07 - 4.26	<0.001
	Poor			6.04	4.18 - 8.72	<0.001
Self-rated health	Limited	15230	240	2.89	1.97 - 4.23	<0.001
	Poor			6.17	4.15 - 9.16	<0.001
Mental health problems	Limited	13974	202	3.05	2.11 - 4.42	<0.001
	Poor			6.19	4.11 - 9.31	<0.001
All above	Limited	11837	186	3.27	2.12 - 5.05	<0.001
	Poor			5.75	3.59 - 9.20	<0.001

The ROC curves displaying sensitivity and specificity of our model including (1) age and sex and (2) age, sex and work ability are presented in Figure 1. Adding work ability improved the performance of the model and suggested that work ability is a good candidate marker for the mortality risk at all time points. The net reclassification indexes are reported in Figure 2. Of all the included variables work ability had the highest NRI. Self-rated health and health behaviors also increased the accuracy of the predicted classification of mortality during the follow-up.

DISCUSSION

For peer review only

The present study using three nationally representative samples suggest that individuals with poor work ability are at a higher risk of mortality than are people with good work ability. This association was observed using a simple, one-item measure of work ability with three response categories. The observed five to six -fold excess risk in the pooled data was not explained by socioeconomic and lifestyle risk factors, self-rated health or mental health problems. The excess risk of mortality in people with poor work ability was also observed in all individual cohorts and thus, our observation may be generalisable over time. Compared to self-rated health, the association between work ability and mortality risk can be considered as robust and large. Our findings support the notion that self-rated health and work ability have only partial overlap with each other and represent different dimensions of health and functional ability. Furthermore, work ability measure clearly improved the prediction of our models compared to other predictors including self-rated health.

It has been shown that poor work ability may predict mortality over relatively long period of time (28), but the majority of research has been conducted on the associations between self-reported work ability and later disability pension or other forms of exit from work (29-32). Most of these previous studies used relatively long and multi-dimensional work ability measures, such as work

For peer review only

ability index, although there are studies showing that the predictive validity of one-item measures may be as good as longer and more comprehensive measures (8).

There are, of course, obvious benefits in measuring work ability with long and comprehensive measures that take into account various medical aspects of the individual´s health and functional capacity, the balance between human resources and work demands, the work community, management, the whole psychosocial work environment and environments outside work life (33). The accuracy and comprehensibility required from the work ability measure depends on the purpose for which it is used, and thus, for example, for granting social benefits the measure of work ability and its influencing factors need to be as valid and reliable as possible. Wide definitions and multidimensional measures are needed specifically when we want to find targets for the interventions aiming to enhance work ability at the individual and organizational level (7).

However, using comprehensive and extensive measurements in research is not always feasible or even necessary. When studying a wide variety of factors determining population health, health care use and health care expenditure, it is not possible to measure all potential factors using detailed and long instruments. Thus, short and economic measures with good predictive validity are needed. The

For peer review only

one-item work ability measure with three response alternative seems to be a promising option for such a measure, capturing the subjective perception of work ability. This measure is simple, easy to understand and can be used as an indicator of work ability based on the conception people have of their own ability to work. A poor work ability may often be related to multiple factors, including multimorbidity and environmental factors and it is, of course, challenging to reduce such psychosocial and physical risks with multifactorial origin. However, multiple risk-adjusted and stepped-care models that provide access to coordinated care of different levels of intensity have been efficiently applied in occupational medicine to manage work disability (2, 34).

The present study has a number of strengths. To the best of our knowledge, with more than 17 000 participants from three cohorts, the present study is a large and comprehensive examination of the association between work ability and mortality. Information on mortality was obtained from the national health register with comprehensive recording system for mortality. Thus, the follow-up was virtually complete and independent of active participation in the studies. The Finnish Causes of Death statistics have been reported to be highly reliable (35). Potential limitations of the study should also be considered. The response rates in individual cohorts ranged from 90 to 68% but the possibility of selection bias in relation to the investigated exposures and outcomes cannot be totally excluded.

For peer review only

Furthermore, the data were limited to participants aged 65 years and younger at baseline. It has been shown that COVID-19 pandemic had a marked impact on all-cause mortality in the European population, after the beginning of 2019 and that excess mortality particularly affected those 65-year-old or older and those with co-morbidities (36). This may have had some small impact on the associations between work ability and mortality in the FinHealth 2017 cohort, but the in the pooled data that effect was probably very small. Although all the sample sizes of the individual cohorts included are quite small compared the whole population it is possible, although highly unlikely that some individuals are included in more than one cohort. However, we do not expect this to be a major source of bias.

Despite these limitations, our findings provide strong population-based evidence to suggest that people with poor self-rated work ability are at increased risk of mortality. Self-rated work ability is a simple and concise measure of health and functional ability with a clear frame of reference and thus it has large potential in screening, epidemiological studies and community-based surveys.

Figure captions

Figure 1. ROC curves of sensitivity and specificity of the model (workability predicting mortality risk) within four follow-up years including age and sex (first row columns) and age, sex and work ability (second row columns).

Figure 2. Net reclassification indexes (NRI) of all included variables predicting mortality risk (the accuracy of the predicted classification of mortality during the four-year follow-up).

Ethics Approval

The two latter surveys were approved by the Ethics Committees of the Finnish Institute for Health and Welfare and the Helsinki (NPHI 8/99) and Uusimaa hospital region (HUS 37/13/00/12016), and all participants gave written informed consent. In the 1970's there was no legally prescribed ethical evaluation concerning research work or informed consent procedures, but the general guidelines concerning medicine were also applicable for research. The subjects invited to the Mini-Finland survey were informed on the use of the data for medical research and participation was interpreted as informed consent for research use of the data.

Contributorship statement

ME, ML, SKa and SKo contributed to the concept and design of the study. ME performed all data analyses. ME wrote the first draft of the manuscript. ME, ML, Ska, AMA, RS and TJ and SKo

For peer review only

contributed to the interpretation of the results, manuscript revision, and approved the final version of the manuscript. ME is responsible for the overall content as guarantor.

Competing interests

The authors declare no competing interests.

Funding

The present study was supported by the Academy of Finland (339390 to ME). The funding bodies had no role in any stage of the research process.

Data sharing statement

Data used in the current study may be obtained from Statistics Finland, and the Finnish Institute for Health and Welfare. Restrictions apply to the availability of these data, which were used under license for this study. For information on accessing the data see: www.stat.fi, and www.thl.fi.

For peer review only

References

1. Bethge M, Spanier K, Kohn S, et al. Self-reported work ability predicts health-related exit and absence from work, work participation, and death: longitudinal findings from a sample of German employees. *Int Arch Occup Environ Health* 2021;94(4):591-9.
2. Aust B, Nielsen MB, Grundtvig G, et al. Implementation of the Danish return-to-work program: process evaluation of a trial in 21 Danish municipalities. *Scand J Work Environ Health* 2015;41(6):529-41.
3. Mittag O, Kotkas T, Reese C, et al. Intervention policies and social security in case of reduced working capacity in the Netherlands, Finland and Germany: a comparative analysis. *Int J Public Health* 2018;63(9):1081-8.
4. Vahtera J, Pentti J, Kivimaki M. Sickness absence as a predictor of mortality among male and female employees. *Journal of Epidemiology & Community Health* 2004;58(4):321-6.
5. Virtanen M, Kivimaki M, Vahtera J, et al. Sickness absence as a risk factor for job termination, unemployment, and disability pension among temporary and permanent employees.[see comment]. *Occupational & Environmental Medicine* 2006;63(3):212-7.
6. Ilmarinen J. Work ability--a comprehensive concept for occupational health research and prevention. *Scand J Work Environ Health* 2009;35(1):1-5.
7. Tuomi K, Huuhtanen P, Nykyri E, et al. Promotion of work ability, the quality of work and retirement. *Occup Med (Lond)* 2001;51(5):318-24.
8. El Fassi M, Bocquet V, Majery N, et al. Work ability assessment in a worker population: comparison and determinants of Work Ability Index and Work Ability score. *BMC Public Health* 2013;13:305.
9. Maddox GL. Some correlates of differences in self-assessment of health status among the elderly. *Journal of gerontology* 1962;17:180-5.
10. LaRue A, Bank L, Jarvik L, et al. Health in old age: how do physicians' ratings and self-ratings compare? *Journal of gerontology* 1979;34(5):687-91.
11. Pijls LT, Feskens EJ, Kromhout D. Self-rated health, mortality, and chronic diseases in elderly men. The Zutphen Study, 1985-1990. *Am J Epidemiol* 1993;138(10):840-8.
12. Idler EL, Russell LB, Davis D. Survival, functional limitations, and self-rated health in the NHANES I Epidemiologic Follow-up Study, 1992. First National Health and Nutrition Examination Survey. *Am J Epidemiol* 2000;152(9):874-83.
13. Haseli-Mashhadi N, Pan A, Ye X, et al. Self-Rated Health in middle-aged and elderly Chinese: distribution, determinants and associations with cardio-metabolic risk factors. *BMC Public Health* 2009;9:368.
14. Hayes AJ, Clarke PM, Glasziou PG, et al. Can self-rated health scores be used for risk prediction in patients with type 2 diabetes? *Diabetes Care* 2008;31(4):795-7.
15. Idler EL, Benyamini Y. Self-rated health and mortality: a review of twenty-seven community studies. *J Health Soc Behav* 1997;38(1):21-37.
16. Idler EL, Kasl S. Health perceptions and survival: do global evaluations of health status really predict mortality? *Journal of gerontology* 1991;46(2):S55-65.

For peer review only

17. Idler EL, Kasl SV, Lemke JH. Self-evaluated health and mortality among the elderly in New Haven, Connecticut, and Iowa and Washington counties, Iowa, 1982-1986. *Am J Epidemiol* 1990;131(1):91-103.
18. Lederer V, Loisel P, Rivard M, et al. Exploring the diversity of conceptualizations of work (dis)ability: a scoping review of published definitions. *J Occup Rehabil* 2014;24(2):242-67.
19. Aromaa A, Heliövaara M, Knekt P, et al. *National Health Examination Surveys in Research*. Helsinki: THL; 2019.
20. Heliovaara M, Aromaa A, Klaukka T, et al. Reliability and validity of interview data on chronic diseases. The Mini-Finland Health Survey. *J Clin Epidemiol* 1993;46(2):181-91.
21. Heistaro S. *Methodology Report. Health 2000 survey. In Publications of the National Public Health Institute, KTL (ed. N. P. H. Institute).* Helsinki: the National Public Health Institute; 2008.
22. Borodulin K, Sääksjärvi K eds. FinHealth 2017 Study – Methods. Helsinki: THL, 2019.
23. Aromaa A, Koskinen S. *Health and functional capacity in Finland. Baseline results of the Health 2000 health examination survey..* Helsinki: National Public Health Institute B3; 2002.
24. Virtanen M, Jokela M, Nyberg ST, et al. Long working hours and alcohol use: systematic review and meta-analysis of published studies and unpublished individual participant data. *BMJ* 2015;350:g7772.
25. Goldberg DP, Gater R, Sartorius N, et al. The validity of two versions of the GHQ in the WHO study of mental illness in general health care. *Psychological Medicine* 1997;27(1):191-7.
26. McCabe CJ, Thomas KJ, Brazier JE, et al. Measuring the mental health status of a population: a comparison of the GHQ-12 and the SF-36 (MHI-5). *Br J Psychiatry* 1996;169(4):516-21.
27. Akritas MG. Nearest-Neighbor Estimation of a Bivariate Distribution under Random Censoring. *Annals of Statistics* 1994;22(3):1299-327.
28. von Bonsdorff. M., Seitsamo J, Ilmarinen J, et al. Work ability in midlife as a predictor of mortality and disability in later life: a 28-year prospective follow-up study. *CMAJ* 2011;184:E235-42.
29. Kinnunen U, Natti J. Work ability score and future work ability as predictors of register-based disability pension and long-term sickness absence: A three-year follow-up study. *Scand J Public Health* 2018;46(3):321-30.
30. Bethge M, Mattukat K, Fauser D, et al. Rehabilitation access and effectiveness for persons with back pain: the protocol of a cohort study (REHAB-BP, DRKS00011554). *BMC Public Health* 2017;18(1):22.
31. Jaaskelainen A, Kausto J, Seitsamo J, et al. Work ability index and perceived work ability as predictors of disability pension: a prospective study among Finnish municipal employees. *Scand J Work Environ Health* 2016;42(6):490-9.
32. Alavinia SM, de Boer AG, van Duivenbooden JC, et al. Determinants of work ability and its predictive value for disability. *Occup Med (Lond)* 2009;59(1):32-7.
33. Ilmarinen J, Tuomi K, Klockars M. Changes in the work ability of active employees over an 11-year period. *Scandinavian Journal of Work, Environment & Health* 1997;23 Suppl 1:49-57.
34. Poulsen OM, Aust B, Bjorner JB, et al. Effect of the Danish return-to-work program on long-term sickness absence: results from a randomized controlled trial in three municipalities. *Scand J Work Environ Health* 2014;40(1):47-56.

For peer review only

35. Lahti RA, Penttila A. Cause-of-death query in validation of death certification by expert panel; effects on mortality statistics in Finland, 1995. *Forensic Sci Int* 2003;131(2-3):113-24.
36. Vestergaard LS, Nielsen J, Richter L, et al. Excess all-cause mortality during the COVID-19 pandemic in Europe - preliminary pooled estimates from the EuroMOMO network, March to April 2020. *Euro Surveill* 2020;25(26).

For peer review only

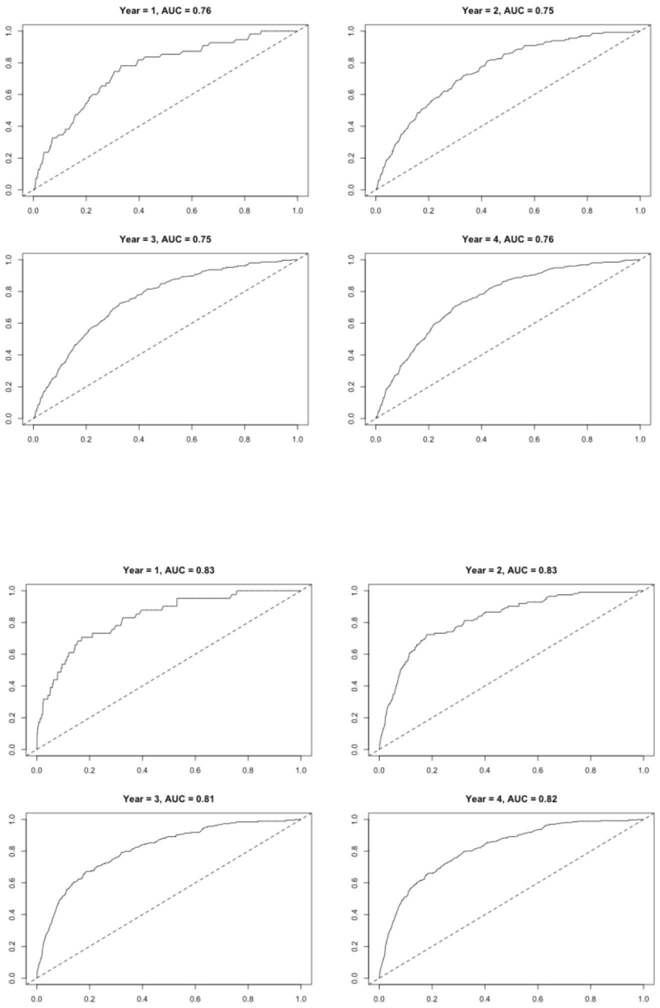

Figure 1. ROC curves of sensitivity and specificity of the model (workability predicting mortality risk) within four follow-up years including age and sex (first row columns) and age, sex and work ability (second row columns).

335x452mm (118 x 118 DPI)

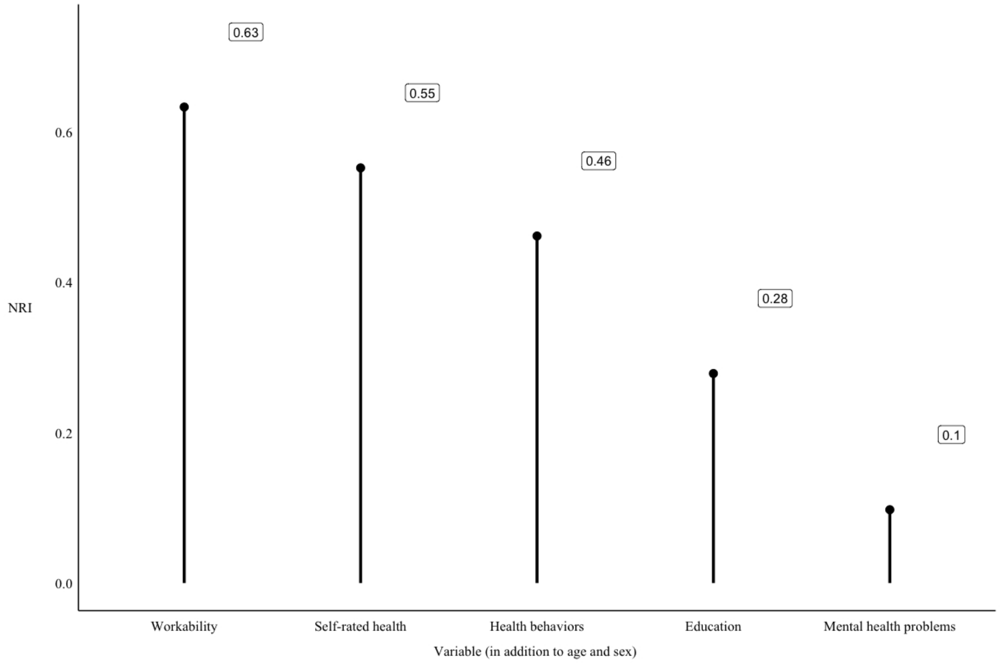

Figure 2. Net reclassification indexes (NRI) of all included variables predicting mortality risk (the accuracy of the predicted classification of mortality during the four-year follow-up).

423x282mm (72 x 72 DPI)

Supplementary table 1. Characteristcs of the Mini-Finland - sample

	Total (N=5897)
**Sex**	
Male	2835 (48.1%)
Female	3062 (51.9%)
**Age_years**	
Mean (SD)	45.8 (9.93)
Median [Min, Max]	45.0 [30.0, 64.0]
**Education**	
Lower	3748 (63.6%)
Intermediate	1684 (28.6%)
Higher	459 (7.79%)
**Alcohol_consumption**	
Low	5397 (95.3%)
High	268 (4.73%)
**Current_smoker**	
No	3047 (51.7%)
Yes	2850 (48.3%)
**Body_Mass_Index**	
Mean (SD)	25.7 (4.02)
Median [Min, Max]	25.2 [16.0, 47.8]
**Mental_health_problems**	
No	4420 (93.8%)
Yes	291 (6.18%)
**Poor_self_rated_health**	
No	2965 (52.3%)
Yes	2700 (47.7%)
**Follow_up_time_from_baseline_years**	
Mean (SD)	3.95 (0.373)
Median [Min, Max]	4.00 [0.00824, 4.00]

Supplementary table 2. Characteristcs of the Health2000 - sample

	Total (N=6723)
**Sex**	
Male	3273 (48.7%)
Female	3450 (51.3%)
**ikä 1.7.2000**	
Mean (SD)	41.1 (12.8)
Median [Min, Max]	42.0 [18.0, 64.0]
**Education**	
Lower	1587 (30.5%)
Intermediate	1882 (36.2%)
Higher	1729 (33.3%)
**Alcohol_consumption**	
Low	5079 (92.4%)
High	416 (7.57%)
**Current_smoker**	
No	4419 (66.0%)
Yes	2276 (34.0%)
**BMI: Painoindeksi**	
Mean (SD)	26.0 (4.70)
Median [Min, Max]	25.3 [12.1, 53.8]
**Mental_health_problems**	
No	5233 (83.7%)
Yes	1021 (16.3%)
**Poor_self_rated_health**	
No	4946 (73.9%)
Yes	1749 (26.1%)
**Short_follow_up_time_from_baseline_years**	
Mean (SD)	3.98 (0.229)
Median [Min, Max]	4.00 [0.0165, 4.00]

Supplementary table 3. Characteristcs of the FinHealth - sample

	Total (N=4556)
**Sex**	
Male	2169 (47.6%)
Female	2387 (52.4%)
**Age_years**	
Mean (SD)	46.1 (12.1)
Median [Min, Max]	47.0 [18.4, 65.0]
**Education**	
Lower	465 (10.2%)
Intermediate	1705 (37.5%)
Higher	2371 (52.2%)
**Alcohol_consumption**	
Low	3335 (73.5%)
High	1201 (26.5%)
**Current_smoker**	
No	1725 (72.6%)
Yes	652 (27.4%)
**Body_Mass_Index**	
Mean (SD)	26.9 (4.99)
Median [Min, Max]	26.0 [15.1, 62.1]
**Mental_health_problems**	
No	4108 (92.2%)
Yes	348 (7.81%)
**Poor_self_rated_health**	
No	3282 (72.2%)
Yes	1261 (27.8%)
**Follow_up_time_from_baseline_years**	
Mean (SD)	3.77 (0.196)
Median [Min, Max]	3.79 [0.146, 3.96]

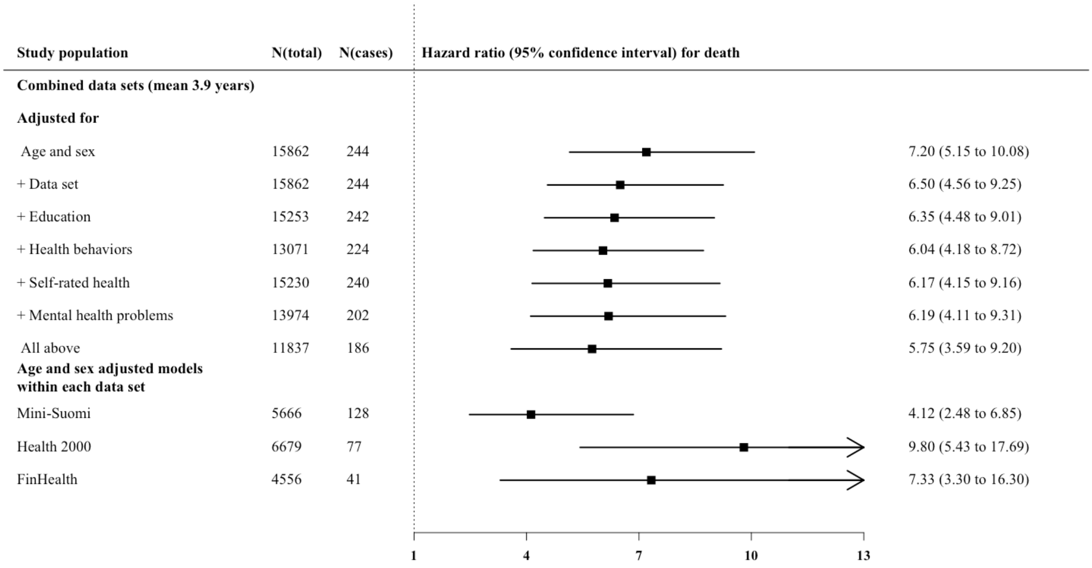

Supplementary figure 1. Associations between poor vs. good workability and mortality risk in pooled and al individual data sets,

For peer review only
